# Effect of different feeding methods on gastrointestinal function in critical patients (DFM-GFC): study protocol for a randomized controlled trial

**DOI:** 10.1186/s13063-022-06807-7

**Published:** 2022-10-20

**Authors:** Guang Yang, Aijing Deng, Bojun Zheng, Jian Li, Yi Yu, Honglian Ouyang, Xin Huang, Hong Chen

**Affiliations:** 1 Department of Intensive Care Unit, Second Clinical College of Guangzhou University of Chinese Medicine, Guangdong Provincial Hospital of Chinese Medicine, 111 Dade Road, Yuexiu District, Guangzhou, Guangdong Province China; 2 Medical Record Department, the Second Affiliated Hospital of Guangzhou University of Chinese Medicine, Guangdong Provincial Hospital of Chinese Medicine, 111 Dade Road, Yuexiu District, Guangzhou, Guangdong Province China; 3 Intensive Care Research Team of Traditional Chinese Medicine, Second Clinical College of Guangzhou University of Chinese Medicine, Guangdong Provincial Hospital of Chinese Medicine, 55 Neihuan West Road, Guangzhou Higher Education Mega Center, Panyu District, Guangzhou, Guangdong Province China

**Keywords:** Critical illness, Enteral nutrition, Feeding method, Gastrointestinal disorders

## Abstract

**Background:**

Enteral nutrition is a major pathway of nutrition for patients requiring critical care. However, it remains unclear whether intermittent or continuous feeding is the better approach, especially after nasogastric enteral nutrition via a gastric tube. Therefore, this randomized controlled clinical study was designed to observe the effects of different methods on critically ill patients.

**Methods:**

Different Feeding Methods on Gastrointestinal Function of Critical patients (DFM-GFC) is a randomized clinical study that will be performed to assess the effects of three feeding methods on critically ill patients. A total of 90 critically ill patients will be equally randomized into three groups: continuous feeding, cyclic feeding, and intermittent feeding. The patients will be administered a gastrointestinal nutrition preparation over 24 h via a gastric tube or over 16 h via an intermittent pump. The primary outcome is the mean duration (days) to reach the caloric goal in each group. Secondary outcomes include the rate of onset of gastric residual, abdominal pressure, the rate of onset pneumonia, and the proportion of individuals achieving the caloric goal. Additionally, the length of intensive care unit (ICU) stay and mortality rate at 28 days post-enrolment will be evaluated.

**Discussion:**

This study will observe the effects of different feeding methods on various parameters, such as the energy target and gastrointestinal motility, in critically ill patients to improve quality of life and reduce the case fatality rate. The purpose of this study is to explore whether there is a more effective, safer and cost-efficient feeding method for the clinical treatment of critically ill patients.

**Trial registration:**

ID: NCT04224883, ClinicalTrials.gov, registered January 9, 2020

**Supplementary Information:**

The online version contains supplementary material available at 10.1186/s13063-022-06807-7.

## Background

The majority of critical patients in the intensive care unit (ICU) require analgesia, sedation, and mechanical ventilation, as they are unable to feed themselves and need enteral nutrition (EN). EN in comparison to parenteral nutrition (PN) is being intensively explored. In the case of critically ill patients, several guidelines recommend initiating EN as soon as possible after ICU admission if the patients are able to eat [1]. These guidelines state than EN feedings via nasogastric or nasointestinal tubes may be delivered by a pump or gravity drip; however, there is no universal standard protocol.

EN can be administered through numerous methods. Three common feeding methods are continuous, intermittent, and cyclic feeding. For example, during continuous feeding, an electric infusion feeding volumetric pump delivers EN at a constant hourly rate 24 h a day. During cyclic feeding, a feeding pump administers EN in < 24 h. During intermittent feeding, EN is pumped through a volumetric pump over 20–60 min every 4–6 h [2]. In intermittent feeding, EN is delivered either by gravity drip of a volumetric pump. Intermittent feeding is not a bolus injection, which is completed in approximately 10 min by manual injection of EN, however this method is rarely used in the ICU.

Some studies suggest that although continuous feeding is well tolerated by patients, it may lead to decreased patient mobility and disrupted circadian cycles because the patient is connected to a feeding pump and the gastrointestinal tract [3, 4]. In contrast to continuous feeding, intermittent feeding is a more physiological mode of delivery. By replicating normal patterns of feeding, intermittent delivery allows a phased supply of hypertonic nutrient loads into the jejunum, which reduces the metabolic demand on the small intestine and prevents excessive accumulation of jejunal fluid [5, 6]. Nevertheless, there can be complications, such as vomiting, aspiration, and fluctuations in blood sugar levels [7]. Overall, cyclic feeding with 6-8 hours of discontinuation enhances a patient’s appetite and/or restores gastric acidity, but only a few studies have focused on this approach. Intermittent feeding at standard intervals allows for greater patient mobility and is considered a physiological method with respect to the cephalic phase of digestion and gut homeostasis [8].

The optimal method of EN delivery in ICU patients remains controversial. In 2017, a critical care nutrition expert panel identified the need for a continuous vs. intermittent feeding trial as one of the “top 10” priority studies to be carried out in the next decade [9]. Moreover, cyclic and intermittent feeding methods are less studied in China. Therefore, we have designed this randomized controlled study to compare the effects of three feeding regimens in critically ill patients. The purpose of our study is to explore whether there is a more effective, safer, and cost-efficient feeding method for critically ill patients.

## Objectives

The primary objective of the DFM-GFC study is to determine whether cyclic or intermittent feeding can extend the duration (days) of achieve caloric goal compared with continuous feeding. The secondary objective of the study is to assess whether the two feeding methods can increase the proportion of patients who achieve the caloric goal, shorten the length of stay in the ICU, and reduce the incidence of feeding complications, including the rates of onset of gastric residua and pneumonia. Abdominal pressure, motilin levels, and mortality in three groups will also be explored.

## Methods

### Design

**Fig. 1:**
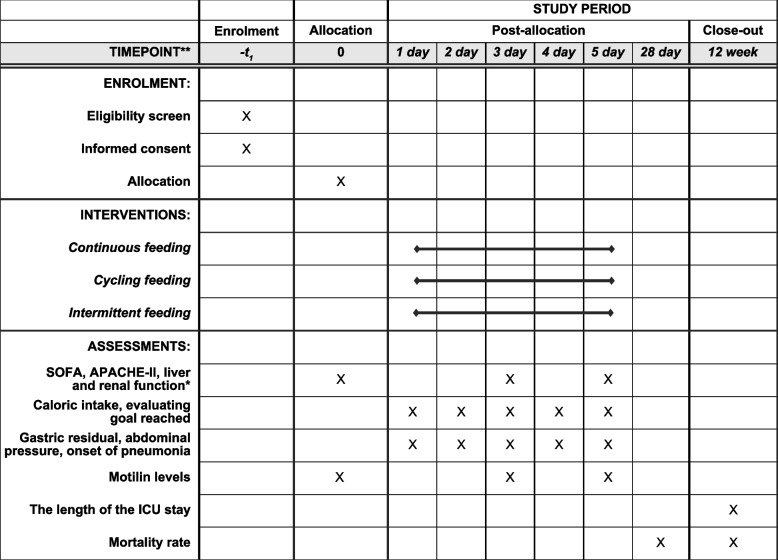
SPIRIT schedule of enrolment, interventions, and assessments. Liver function includes alanine aminotransferase (U/L), aspartate aminotransferase (U/L), total bilirubin (μmol/L), direct bilirubin (μmol/L), and albumin (g/L). Renal function includes blood creatinine (mmol/L) and urea (mmol/L)

This is a prospective randomized controlled study. There are three parallel groups: a continuous feeding group, cyclic feeding group, and intermittent feeding group. The trial protocol was written according to the Standard Protocol Items: Recommendations for Interventional Trials Statement (SPIRIT). A SPIRIT table is provided in Fig. 1, and a SPIRIT checklist is included in Additional file 1.

### Allocation and blinding

A random number table generated by SPSS 20.0 software (IBM Co., Armonk, NY, USA), using the randomization method, will be employed to assign the participants to each group at a ratio of 1:1:1. Patients will be enrolled during allocation according to the sequence of ICU admission. For masking, random sequences and group assignments will be sealed in opaque envelopes. As this study is a comparative assessment of treatment modalities, blinding will not be feasible. Masking related to the participant treatment arm allocation and to the treatment team for the baseline and follow-up assessments will be applied for the assessment team, data manager, and statistician.

### Participants

Three separate cohorts of patients admitted to the ICU of Guangdong Hospital of Chinese Medicine who satisfy the inclusion/exclusion criteria outlined below will be recruited for this study. Before recruitment, the research team will prepare and publish recruitment advertisements and hold consultation meetings with doctors specializing in critical care in the hospital.

### Inclusion criteria

The following inclusion criteria will be applied: participants (1) aged 18–80 years, (2) with Acute Physiology and Chronic Health Evaluation (APACHE)-II ≥ 15 [10], (3) in whom EN can be used after evaluation, (4) with an expected ICU stay of > 48 h, and (5) patients or family members signed informed consent.

### Exclusion criteria

The following exclusion criteria will be applied: participants (1) required to fast clinically, such as those with digestive tract perforation, bleeding, or gastrointestinal tract surgery; (2) who are allergic to EN preparations; (3) have unstable hemodynamics; (4) who are unwilling to participate in the trial or cannot cooperate with the treatment, and (5) who are pregnant and lactating.

### Interventions

Patients will be randomized into three arms: a continuous feeding group, cyclic feeding group, and intermittent feeding group. In the continuous group, an electric infusion feeding pump will deliver EN at a constant hourly rate of 24 h/day. The critical patients who are randomized to the cyclic feeding group will receive the EN preparation for 16 h via continuous pumping through the stomach tube every day. The initial pumping speed of the two groups will be calculated as the average pumping volume of the total enteral nutrition in 1 day. For example, if the total daily intake is 1200 mL, the initial pumping speed of the continuous group will be 50 mL/h, which of cyclic is 75 mL/h. The gastric residual volumes (GRV) will be checked every 4 h. If it is not tolerable, the pumping speed will be reduced by half of the original speed. Feeding intolerance will be defined as GRV > 250 mL; EN will be stopped and reassessed after 4 hours if GRV > 500 mL. The critical patients who are randomized to the intermittent feeding group will be administered the EN preparation through four meals every day (at 08:00, 12:00, 18:00, and 22:00). In the intermittent group, the daily amount of feeding will be divided into four meals, and each meal will be pumped through the stomach tube within 60 min or 120 min. The following EN preparation pumping scheme will be used. If the volume of each meal is less than or equal to 250 mL (≤ 250 mL), it will be delivered within 60 min; if the volume is greater than 250 mL (> 250 mL), it will be delivered within 120 min. If the total daily intake is 1200 mL, the initial pumping speed will be 150 mL/h. GRV will be checked before each intermittent feeding. If it is not tolerable, the pumping speed will be reduced by half of the original speed. If GRV > 500 mL, EN will be stopped and reassessed after 4 hours. All feeding will be started within 24 h of ICU admission. An EN suspension ((SP, Peptisorb), Nutricia Pharmaceuticals (Wuxi, Jiangsu, China) Co. Ltd) will be used, with a calorie content of 1 kcal/mL and a thermal nitrogen ratio of 133:1. Each bottle will contain 500 mL of nutrition preparation consisting of 61.5 g carbohydrate, 20.0 g protein, and 19.45 g fat (Wuxi). The nutritional preparations for the three groups of patients will be pumped into the nasal feeding tube through a volumetric pump (brand: Braun, origin: Melsungen AG, Germany). The treatment course will be 5 days (Table 1).

**Table 1:** EN pumping speed scheme

Time		Continuous group	Cyclic group	Intermittent group
00:00	Pumping speed	50 ml/L		
	Check GRV	Check		
04:00	Pumping speed	50 ml/L		
	Check GRV	Check		
08:00	Pumping speed	50 ml/h	75 ml/h	150 ml/h
	Check GRV	Check	Check	Check
12:00	Pumping speed	50 ml/h	75 ml/h	150 ml/h
	Check GRV	Check	Check	Check
16:00	Pumping speed	50 ml/h	75 ml/h	
	Check GRV	Check	Check	
18:00	Pumping speed	50 ml/h	75 ml/h	150 ml/h
	Check GRV			Check
20:00	Pumping speed	50 ml/h	75 ml/h	
	Check GRV	Check	Check	
22:00	Pumping speed	50 ml/h	75 ml/h	150 ml/h
	Check GRV			Check

*GRV* Gastric residual volumes; the pumping speed will be adjusted according to GRV as follows: ≤ 250 mL, hold and recheck after 4 hours; > 250 mL: decrease to half of the original speed and recheck after 4 hours; and > 500 mL: stop and recheck after 4 h

The researchers who implement the intervention measures will receive unified training. Specialized researchers will supervise the compliance of the pumping mode by checking the execution of the doctors’ orders. Relevant concomitant care will be prohibited during the trial.

### Criteria for drop-out

The following criteria for drop-out will be used: participants (1) who cannot tolerate the nutrition preparation, resulting in diarrhea, severe abdominal distension, and other complications, and (2) who have an automatic withdrawal request submitted by either themselves or their family members at any time.

### Outcomes

#### Primary outcome

(1) The primary outcome is the mean duration (days) of achieve the caloric goal in every group. The caloric goal of 25 kcal/kg (ideal body weight) for caloric need was calculated by a single nutritionist. The duration will be the first 5 days after the intervention.

#### Secondary outcome

Secondary outcomes include the following: (1) the rate of onset of gastric residual (%) (defined as volume >500 mL) among the three groups; (2) abdominal pressure, (mmHg) as measured through the indirect bladder pressure, whereby the bladder urine will be emptied in the supine position, and 50 mL saline will be poured into the balloon catheter to the pubic symphysis as the base point, keeping the piezometric tube perpendicular to the ground to obtain indirect abdominal pressure; (3) the rate of onset of pneumonia in each group (%), as defined by two of the following clinical criteria, fever (> 38.3 °C) or hypothermia (≤ 36.0 °C) and leucocytosis (> 10×10^9^ cells/L) or leucopenia (≤ 4×10^9^ cells/L) and purulent tracheal aspirate or sputum. These factors are associated with the appearance of a new infiltrate or changes in the existing infiltrate on chest X-ray [11]; (4) the proportion (%) of individuals who achieve the caloric goal. The caloric goals using 25 kcal/kg (ideal body weight) for caloric need were calculated by a single nutritionist; (5) motilin levels before and 5 days after intervention; (6) the length of the ICU stay (in days) up to 12 weeks; and (7) the ICU mortality rate (%) at 28 days after intervention and survival analysis up to 12 weeks (Fig. 1). Follow-up by visit or telephone will be conducted at 28 days and 12 weeks after the intervention.

#### Sample size

The sample size was calculated based on the duration required to achieve the caloric goal. Because there are few studies about cyclic feeding, we referred to the studies on intermittent and continuous groups. It was assumed that the time to achieve the caloric goal would be consistent with earlier RCTs [12]: 4 days (intermittent feeding group) and 3 days (continuous feeding group). Twenty-six evaluable patients per group will provide 90% power for detection at a 5% two-sided significance level. Therefore, the total sample size will be 84. Based on the sample sizes in previous studies and an estimated dropout rate extrapolated from feasibility data of 8–10% [13], we expect that 30 patients will be enrolled in each arm and that the total sample size will be at least 90.

### Consent or assent

The patients who fulfill the study requirements will be offered a consent form consisting of the details, such as the name of the study, registered information, research background, how the study will be conducted, what the participants should do in the study, inclusion/exclusion criteria, treatment plans and obligations, putative drug-related side effects, and expenses during participation. If the patient is not conscious, his or her authorized agent or family member can provide informed consent. The patients’ personal medical data will be kept confidential. Participation in this study will be voluntary, and other treatment options will be offered to the patients who did not participate or dropped out. Informed consent will be obtained from the patients to utilize their personal and medical information in this study. To protect the privacy of the subjects, data processing will be performed anonymously, and informed consent will be obtained from each patient or statutory agent before initiation of the clinical trial.

### Data collection and management

The Second Affiliated Hospital of Guangzhou University of Chinese Medicine will be in charge of the data management and statistical analysis in this study. The clinical observations do not participate in statistical analysis. The study personnel will undergo training sessions on data collection and will be individually tested regarding data entry as well as outcome assessments before initiation of the trial. The study data will be collected and managed using SPSS 20.0. A follow-up visit or telephone follow-up will be conducted at 28 days and 12 weeks after the intervention.

An independent data safety monitoring board (DSMB) will receive the trial data from the biostatistician and choose to either continue the study as planned, change the study protocol, or stop the trial early due to harm.

The participants’ medical records will be maintained at the hospital, and the investigator, research authority, and ethics committee will be allowed access to this information. Any public report on the results of this study will not disclose the participants’ personal identities. Every effort will be made to protect the privacy of the participants’ personal medical data, according to law. Personal and medical information will be kept confidential in a safe and reliable location. At any time, the participants will be able to request access to their personal information (such as their address and contact information) and modify it if necessary.

The clinical specimen department of Guangdong Academy of Chinese Medicine will carry out the unified management and preservation of collected blood specimens. Motilin levels will be determined by ELISA at the Laboratory of Critical Care Medicine of Guangdong Academy of Chinese Medicine.

### Statistical analysis

All statistical analyses will be performed using SPSS 20.0 software. Measurement data will be expressed as the mean ± standard deviation. Normality tests and homogeneity tests of variance will also be performed. In cases of normal distribution and homogeneity of variance, a *t* test will be conducted; otherwise, a non-parametric test will be employed. If the measurement data conform to a normal distribution, an independent sample *t* test will be applied between two groups; one-way analysis of variance (ANOVA) will be utilized between the three groups. In the case of non-normal distribution, two groups will be compared using the Mann–Whitney *U* test, and the three groups will be compared with the Kruskal–Wallis test. Enumeration data between two and three groups will be compared using chi-square/Crosstab statistical analysis, and the results will be expressed as the frequency constituent ratio (%). *P* < 0.05 will indicate statistical significance.

For patients who die or are lost to follow-up, their observation time will be censored at the time of death or date of last contact. For patients with early withdrawal who are lost to follow-up, intention-to-treat analysis (ITT) will be applied. For the second endpoint (6), cumulative proportion survival curves according to Kaplan–Meier analysis will be obtained for each group and compared with the log-rank test. To explore the group effect on the time to the event of composite outcome, with adjustment for baseline covariates, the Cox regression model will be used.

The trial will be prematurely paused or closed by the DSMB to evaluate safety information from the study or if there is evidence of harm in the study. The principal investigator (PI) will be able to access these interim results.

### Oversight and monitoring

The coordinating center, steering committee, and endpoint adjudication committee’s responsibilities will be assumed by the ethics committee of Guangdong Hospital of Chinese Medicine, with review of the data for at least half a year.

Monitoring will be performed by an independent DSMB consisting of three members with expertise in critical care medicine, clinical trial methodology, and biostatistics. The DSMB will receive the trial data from the biostatistician and choose to either continue the study as planned, change the study protocol, or stop the trial early due to harm. The DSMB will submit the experimental data to the ethics committee of Guangdong Hospital of Traditional Chinese Medicine for review at least once every 6 months. Related data from each DSMB meeting will be retained in a secured file for inspection by regulatory authorities.

### Adverse events and injury

Although adverse events (AEs) of the intervention measures in this study will be rare, any AEs that occur in subjects during clinical observation might be related to the drug of interest. The AE report form will be completed during the trial. The time of occurrence, severity, duration, actions taken, and outcomes of AEs will be recorded, and those occurring during follow-up will be reported to the sponsor in a timely manner. Furthermore, serious AEs will be reported simultaneously to the adverse drug reaction (ADR) monitoring center of the local authority within 24 h and the sponsor. The study team will prioritize unpaid examination and treatment according to the regulations of the ethics committee. Auditing trial conduct will occur once a year.

### Protocol amendments

The process will be independent from the investigators and the sponsor. If the research plan needs to be modified, the ethics committee will be provided with relevant explanatory materials and an application, and the modification will only be carried out after approval.

## Discussion

Although guidelines state that EN should be given by a pump or gravity drip, there is no agreement on whether continuous pumping should be applied or on the duration of pumping. The DFM-GFC study will explore the impact of three common EN delivery methods (continuous, intermittent, cyclic) on heat entrapment, gastrointestinal complications, and the occurrence of aspiration and aspiration pneumonia. In addition, costs associated with EN delivery (equipment use and complications) will be compared. Although the incidence of complications will not decline significantly, the cost of treatment will be lower if the intermittent pumping and cyclic pumping feeding method are accomplished in less time than the continuous pumping feeding method.

Current guidelines often compare continuous feeding to intermittent feeding (done either via manually set gravity rate or volumetric pump) [1]. The number of volumetric pumps available may vary from institution to institution. However, the use of a volumetric pump in place of manual infusion (gravity drip) for intermittent feedings allows for more consistent feeding. All three feeding methods, continuous, intermittent, and cyclic are commonly used in hospitals [14, 15]. Nonetheless, with the application of a volumetric pump, manual injection is no longer used for intermittent feeding, and the time of each injection can also be extended to several hours. Continuous feeding, intermittent feeding, and cyclic feeding have become three common feeding methods. Some studies [13, 16, 17] have demonstrated that continuous pumping does not improve blood sugar levels, stomach retention, or the occurrence of ventilator-associated pneumonia.

According to an individual’s physiology and habits, it is necessary to rest during the inter-meal time, the physiologically appropriate number of meals per day for humans is an enigma. According to Homer, only two, rather than three, meals were eaten per day in ancient Greece. During sleep, peristalsis of the gastrointestinal tract is slowed, and secretion of digestive juices is reduced. Some studies [18] have demonstrated that there is no difference in the amount of water in the small intestine and the amount of hormones secreted by the small intestine using continuous pumping and bolus through a gastric tube in healthy individuals. Therefore, is intermittent eating the result of physical conditions and social behavior or the physiological needs of organs?

A previous study [19] has found that intermittent bolus feeding enhances muscle protein synthesis to a greater extent than continuous feeding by eliciting a pulsatile pattern of amino acid- and insulin-induced translation initiation. Moreover, it increases difficulty in moving or turning [20]. Overall, continuous pumping is not a physiological condition. Although intermittent feeding can be cost-effective for patients, it is unclear whether continuous pumping is optimal in terms of treatment effectiveness and complications. Therefore, this study will compare the effects of continuous feeding, cyclic feeding, and intermittent feeding in critically ill patients, including complications such as vomiting, diarrhea, length of stay in the ICU and increased hospitalization costs due to aspiration pneumonia. In summary, this study will reveal cyclic feeding or intermittent feeding is the optimal treatment for EN in critically ill patients and will serve as a basis for the development of EN guidelines.

## Trial status

The systematic reviews of quantitative and qualitative literature have been completed and published. Enrolment is ongoing. Recruitment started in July 2020 is expected to conclude in December 2023. Target enrolment for the study is 90 participants. Protocol version: V2.0, 01092020.

## Supplementary Information

**Additional file 1.**

## Data Availability

Data results from this randomized controlled study will be unavailable at the time of publication. Individual participant data will be available upon request.

## Abbreviations

ICU: Intensive care unit
EN: Enteral nutrition
PN: Parenteral nutrition
APACHE-II: Acute Physiology and Chronic Health Evaluation-II
GRV: Gastric residual volume
AE: Adverse event
ADR: Adverse drug reaction
DSMB: Data safety monitoring board
ELISA: Enzyme linked immunosorbent assay
